# Fosfomycin monotherapy for the treatment of a complicated prostatic abscess caused by KPC-producing carbapenem-resistant *Escherichia coli*

**DOI:** 10.1128/asmcr.00112-26

**Published:** 2026-09-15

**Authors:** Nguyen Thi Tuyen, Nguyen Sy Thau, Pham Thi Thanh Nhan, Nguyen Van Tuan, Nguyen Trong The

**Affiliations:** 1 Vietnamese-German Center for Medical Research (VG-CARE), 108 Military Central Hospital 625280, Hanoi, Vietnam; 2 Institute of Clinical Infectious Diseases, 108 Military Central Hospital 625280, Hanoi, Vietnam; 3 Department of Functional Exploration, 108 Military Central Hospital 625280, Hanoi, Vietnam; Vanderbilt University Medical Center, Nashville, Tennessee, USA

**Keywords:** fosfomycin, prostatic abscess, KPC, carbapenem-resistant, *Escherichia coli*

## Abstract

**Background:**

Prostatic abscess caused by carbapenem-resistant *Escherichia coli* (CREC) is an uncommon but serious infection with limited therapeutic options, particularly when prolonged antimicrobial therapy and oral step-down treatment are required. Evidence supporting fosfomycin monotherapy for prostatic abscess caused by CREC remains scarce.

**Case Summary:**

A 67-year-old man with diabetes mellitus and prolonged corticosteroid exposure presented with severe sepsis secondary to a multiloculated prostatic abscess measuring 57.2 × 38.7 × 49.4 mm. Urine and abscess cultures yielded KPC-producing CREC susceptible to fosfomycin (MIC = 0.25 μg/mL) and amikacin. Because access to newer β-lactam/β-lactamase inhibitor agents was limited, treatment was initiated with intravenous fosfomycin plus amikacin. Amikacin was discontinued after 2 days because of nephrotoxicity, and therapy was successfully continued with intravenous fosfomycin monotherapy following transperineal ultrasound-guided abscess drainage. The patient received intravenous fosfomycin 6 g every 8 h for 21 days, followed by oral fosfomycin calcium 3 g every 24 h for 30 days, with complete clinical and radiological resolution, and no relapse during 6 months of follow-up.

**Conclusion:**

Although the initial brief course of amikacin may have contributed to early bacterial clearance, the majority of treatment was administered as fosfomycin monotherapy after adequate source control. This case suggests that antibiotic susceptibility test-guided fosfomycin monotherapy, combined with adequate drainage, is a feasible treatment for prostatic abscesses caused by CREC, particularly in resource-limited settings.

## INTRODUCTION

A prostatic abscess is an uncommon but severe complication of acute bacterial prostatitis that may progress to sepsis and often requires prolonged antimicrobial therapy, together with surgical or percutaneous drainage in selected cases (1). Patients with severe infections should receive prompt empirical broad-spectrum intravenous antibiotics, often as combination therapy, followed by transition to oral agents whenever feasible to reduce hospitalization. The increasing prevalence of multidrug-resistant organisms, particularly carbapenem-resistant *Enterobacterales* (CRE), has created substantial therapeutic challenges during both the initial intravenous treatment phase and subsequent oral step-down therapy (2). In particular, infections caused by *Klebsiella pneumoniae* carbapenemase (KPC)-producing strains are associated with substantial mortality and limited therapeutic options (2, 3). Although newer β-lactam/β-lactamase inhibitor combinations such as ceftazidime–avibactam have improved outcomes, their availability and high cost restrict their use in many regions and circumstances (4). Moreover, antibiotic selection remains challenging because many agents have poor penetration into prostatic tissue (5).

Fosfomycin has re-emerged as a promising option owing to its unique mechanism of action, retained activity against many carbapenem-resistant *Escherichia coli* (CREC) isolates, and favorable pharmacokinetics (5, 6). Importantly, fosfomycin is available in both intravenous and oral formulations, allowing a seamless transition from intensive inpatient treatment to outpatient oral therapy. In addition, it achieves therapeutic concentrations in inflamed prostatic tissues, supporting its use in deep-seated urogenital infections (7, 8).

Although some studies have supported the use of intravenous fosfomycin monotherapy for complicated urinary tract infections, including acute pyelonephritis (9), and oral fosfomycin for chronic bacterial prostatitis (10), evidence for its use as monotherapy in the treatment of prostatic abscesses remains scarce. Herein, we report a case of complicated prostatic abscess caused by KPC-producing CREC that was successfully treated with fosfomycin monotherapy combined with transperineal ultrasound-guided abscess drainage.

## CASE PRESENTATION

**Fig 1:**
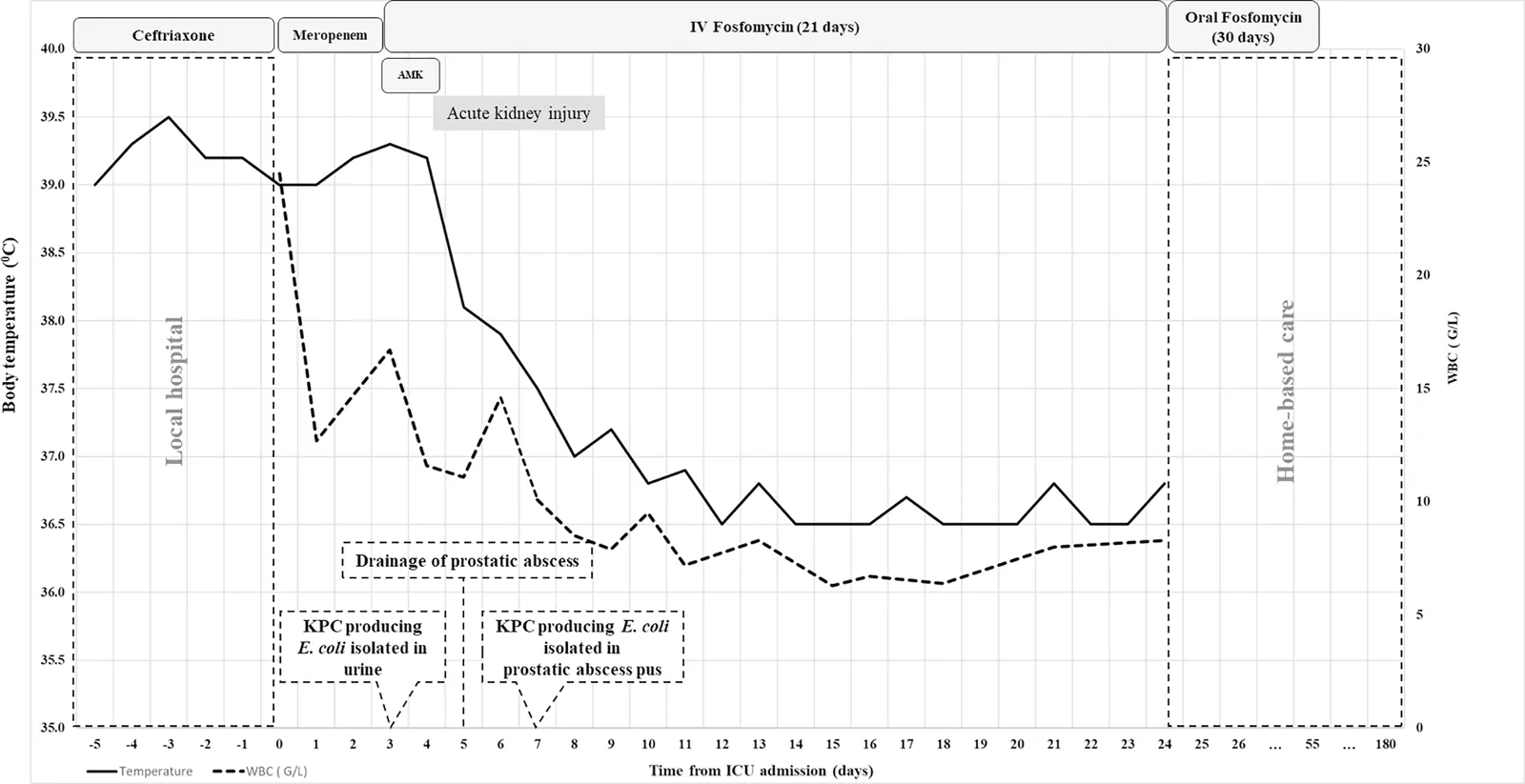
Timeline of clinical course in the present case. The figure illustrates the key diagnostic investigations and interventions. The solid line (temperature) corresponds to the left *y*-axis, whereas the dotted line (WBC count) corresponds to the right *y*-axis. AMK, amikacin.

**Fig 2:**
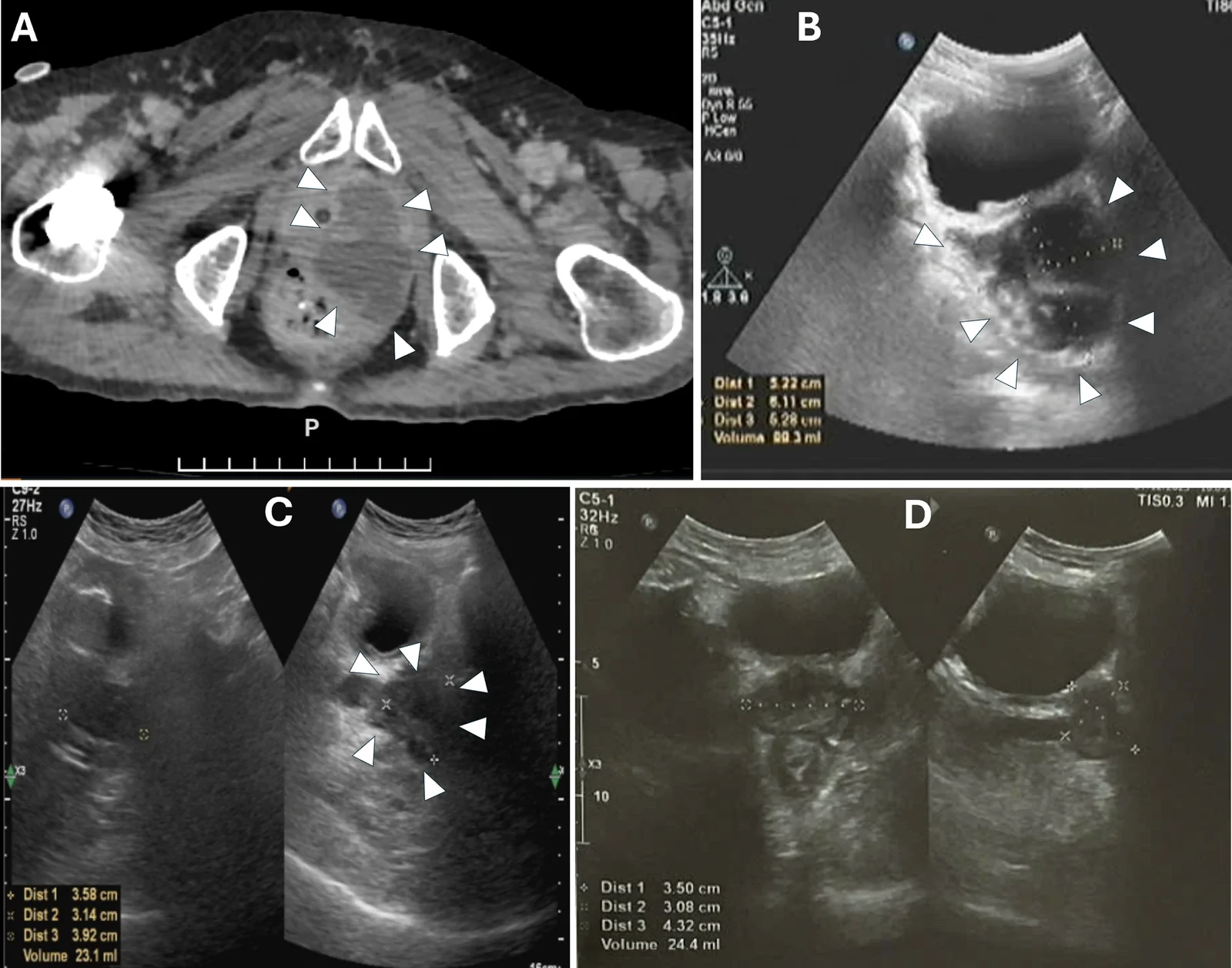
Computed tomography and ultrasound imaging of prostatic abscess due to *E. coli*. (**A**) Axial CT scan image at admission demonstrated multiple hypodense fluid collections within the prostatic parenchyma, coalescing into a large multiloculated prostatic abscess, with the largest confluent cavity measuring 57.2 × 38.7 × 49.4 mm. (**B**) Abdominal ultrasonography at admission showed an enlarged prostate (prostate volume approximately 88 mL) with multiple ill-defined hypoechoic lesions consistent with prostatic abscesses. (**C**) Follow-up ultrasonography on hospital day 20 demonstrated further reduction in abscess size (prostate volume approximately 23 mL) with minimal residual hypoechoic foci. (**D**) Follow-up ultrasonography performed 21 days after discharge demonstrated near-complete radiological resolution of the prostatic abscess (prostate volume approximately 24 mL).

A 67-year-old man with type 2 diabetes mellitus, hypertension, chronic hepatitis B, and Cushing’s syndrome secondary to prolonged corticosteroid exposure presented with severe sepsis. Four months before admission, he had been hospitalized for surgical site infection following hemiarthroplasty and had received prolonged broad-spectrum antimicrobial therapy complicated by ischemic stroke with residual neurological deficits. Five days before admission, he developed high-grade fever with rigors and dysuria that failed to respond to intravenous ceftriaxone (2 g every 24 h) initiated at a local hospital. Progressive clinical deterioration prompted transfer to the intensive care unit (ICU). On presentation, he had a Glasgow Coma Score of 10, requiring supplemental oxygen via a nasal cannula, and had a SOFA score of 7. He was febrile (38.5°C) and laboratory investigations demonstrated leukocytosis with a white blood cell (WBC) count of 24.9 × 10^9^/L (reference range: 4.0–10.0^9^/L) and neutrophil predominance (92%, reference range: 40–74%), elevated C-reactive protein (CRP) level of 229 mg/L (reference range: <5 mg/L), and procalcitonin (PCT) level of 122 ng/mL (reference range: <0.05 ng/mL) (Fig. 1). Acute urinary retention required bladder catheterization, which drained cloudy urine, and urinalysis revealed leukocyturia, erythrocyturia, and a positive nitrite test. Abdominal computed tomography (CT) (Fig. 2A) and ultrasonography (Fig. 2B) revealed a large multiloculated prostatic abscess, with the largest confluent cavity measuring 57.2 × 38.7 × 49.4 mm. A diagnosis of sepsis secondary to a prostatic abscess was established, and empiric intravenous meropenem (1 g every 8 h) was initiated.

On hospital day 3, the midstream urine culture obtained on ICU admission grew KPC-producing CREC. Antimicrobial susceptibility testing demonstrated retained susceptibility to amikacin, ceftazidime–avibactam, and imipenem–cilastatin–relebactam (Table 1). The fosfomycin minimum inhibitory concentration (MIC) was 0.25 μg/mL, as determined by E-test (bioMérieux, Marcy-l’Étoile, France) on Mueller-Hinton agar supplemented with 25 μg/mL glucose-6-phosphate and interpreted as susceptible according to the CLSI M100 urinary *E. coli* breakpoint (≤64 µg/mL). Carbapenemase testing using the NG-Test CARBA 5 assay confirmed KPC production, whereas OXA-48-like, IMP, NDM, and VIM carbapenemases were not detected (Table 1). A set of blood cultures obtained on ICU admission showed no growth. Due to limited access to novel β-lactam/β-lactamase inhibitor (BL/BLI) agents, antimicrobial therapy was switched to intravenous amikacin (15 mg/kg/day) in combination with intravenous fosfomycin (6 g every 8 h). On hospital day 5, amikacin was discontinued because of worsening renal function (serum creatinine increased to 2.2 mg/dL, reference range: 0.67–1.18 mg/dL), and treatment was continued with intravenous fosfomycin monotherapy. Concurrent transperineal ultrasound-guided aspiration yielded approximately 30 mL of thick, milky, purulent material. Culture of the aspirated pus confirmed the presence of KPC-producing CREC, consistent with the urine isolate.

**TABLE 1:** Antimicrobial susceptibility profile and detection of carbapenemase in *Escherichia coli* isolate

Antimicrobial agent or carbapenemase	MIC (μg/mL) or result	CLSI interpretation
Antimicrobial agent^*a*^		
Ceftazidime	32	Resistant (R)
Cefepime	≥32	Resistant (R)
Piperacillin/tazobactam	≥128	Resistant (R)
Ciprofloxacin	≥4	Resistant (R)
Levofloxacin	≥8	Resistant (R)
Amikacin	4	Susceptible (S)
Tobramycin	8	Resistant (R)
Aztreonam	≥64	Resistant (R)
Imipenem	≥16	Resistant (R)
Meropenem	≥16	Resistant (R)
Ceftazidime/avibactam	0.25	Susceptible (S)
Ceftolozane/tazobactam	8	Resistant (R)
Imipenem/relebactam	≤0.25	Susceptible (S)
Colistin	0.5	Intermediate (I)
Fosfomycin	0.25	Susceptible (S)
Trimethoprim/sulfamethoxazole	8/152	Resistant (R)
Carbapenemase**^*b*^**		
KPC	Detected	
OXA-48-like, IMP, VIM, NDM	Not detected	

^*a*^ Fosfomycin susceptibility testing was performed using E-test (bioMérieux) on Mueller-Hinton agar supplemented with 25 μg/mL glucose-6-phosphate. Vitek 2 Gram-Negative Susceptibility Card AST-N443 (bioMérieux) was used for all other susceptibility testing. Interpretive criteria were based on CLSI M100 (35th edition, 2025).

^*b*^ IMP, imipenemase metallo-β-lactamase; KPC, *Klebsiella pneumoniae* carbapenemase; NDM, New Delhi metallo-β-lactamase; OXA-48-like, oxacillinase-48-like; VIM, Verona integron-encoded metallo-β-lactamase. Carbapenemase production was confirmed using the NG-Test CARBA 5 lateral flow immunoassay (NG Biotech, Guipry, France).

The patient demonstrated gradual clinical improvement, with defervescence by hospital day 6 and progressive reduction in inflammatory markers (PCT decreasing to 43.9 ng/mL by hospital day 6 and CRP decreasing to 35.7 mg/L by hospital day 24). Renal function recovered within 4 days after amikacin discontinuation, with the serum creatinine level decreasing to 1.01 mg/dL on hospital day 9. The patient completed a 21-day course of intravenous fosfomycin and was discharged home on hospital day 24. At discharge, repeat ultrasonography demonstrated a marked reduction in prostate volume (approximately 23.1 mL), with residual heterogeneous echotexture and no discrete fluid collections (Fig. 2C). Oral fosfomycin calcium (3 g every 24 h) was prescribed for an additional month after discharge. Follow-up ultrasonography performed 21 days after discharge demonstrated near-complete radiological resolution (Fig. 2D), and pelvic CT scan performed 8 weeks after discharge confirmed complete resolution of the prostatic abscess, with no residual collections. At the 6-month follow-up, the patient remained asymptomatic and afebrile with no evidence of prostatitis relapse.

## DISCUSSION

Prostatic abscesses caused by CREC represent a major therapeutic challenge, particularly in immunocompromised patients with prior healthcare exposure and broad-spectrum antibiotic use. Our patient had multiple risk factors for invasive CREC infection, including diabetes mellitus, chronic corticosteroid exposure, prolonged hospitalization, and previous antimicrobial therapy. These factors likely contributed to the colonization and subsequent infection with KPC-producing CREC. The present case highlights several clinically important aspects, including the significance of prompt source control, pharmacologic rationale for fosfomycin use in prostatic infections, and potential role of fosfomycin monotherapy as a novel BL/BLI-sparing strategy in carefully selected patients.

The management of prostatic abscesses requires prolonged targeted antimicrobial therapy and adequate source control. Drainage is generally recommended in patients with multiloculated abscesses, persistent sepsis, or lesions >2 cm (1, 11). In our case, transperineal ultrasound-guided aspiration likely contributed substantially to the favorable outcomes by reducing the bacterial burden and improving antibiotic penetration.

Treatment selection was particularly challenging because the isolate demonstrated KPC-mediated carbapenem resistance. Although ceftazidime–avibactam is considered the preferred therapy for KPC-producing CREC, its availability and cost remain limiting factors in many settings (4). Aminoglycosides and polymyxins are also constrained by toxicity and uncertain prostatic penetration (12). In our patient, amikacin was discontinued after 2 days because of nephrotoxicity, emphasizing the limitations of aminoglycoside-based regimens in elderly patients with multiple comorbidities. Fosfomycin represents a rational alternative because of its retained activity against many CREC isolates, its unique mechanism of action, and its favorable pharmacokinetic profile (6, 13, 14). Importantly, fosfomycin achieves therapeutic concentrations within inflamed prostatic tissues, supporting its use in deep-seated urogenital infections (7, 8). Although fosfomycin is commonly administered as part of combination therapy for severe CREC infections, successful continuation as monotherapy was observed in our patient, following adequate drainage and rapid clinical improvement. The brief course of amikacin may have contributed to early bacterial clearance; however, the patient received the vast majority of therapy with fosfomycin monotherapy after amikacin discontinuation. The low fosfomycin MIC, absence of bacteremia, and prolonged treatment duration may have contributed to the favorable outcome.

The availability of oral fosfomycin additionally provides an important advantage by allowing prolonged outpatient therapy after hospital discharge. This approach may be particularly valuable in settings with a high prevalence of CREC. Nevertheless, resistance emergence during fosfomycin therapy remains a concern, particularly in infections with high bacterial burden (14). Moreover, existing evidence supports the use of fosfomycin for prostatitis caused by *E. coli*, whereas its use for infections caused by other gram-negative organisms remains limited because many of these species frequently harbor fosA genes that can inactivate fosfomycin and increase the risk of clinical failure (2). Agar dilution supplemented with glucose-6-phosphate is the CLSI reference method for fosfomycin susceptibility testing of *E. coli*. In our laboratory, fosfomycin susceptibility was assessed by *E*-test on glucose-6-phosphate-supplemented Mueller-Hinton agar, which is not the reference method but has been shown to reliably identify fosfomycin-susceptible *E. coli* urinary isolates (15). Therefore, fosfomycin monotherapy should be considered cautiously and preferably in carefully selected patients with confirmed susceptibility and adequate source control measures. Although limited by its single-case design, this report adds to the growing evidence supporting fosfomycin-based strategies for CREC-associated prostatic infections and highlights fosfomycin monotherapy as a potential carbapenem-sparing option under selected clinical circumstances.

### Conclusion

This case suggests that prolonged fosfomycin monotherapy following early discontinuation of amikacin, guided by susceptibility testing combined with adequate abscess drainage, may be an effective treatment option for prostatic abscess caused by CREC. Fosfomycin may represent a valuable carbapenem-sparing alternative, particularly in resource-limited settings, or when other agents are limited by toxicity. Further studies are needed to better define the role of fosfomycin monotherapy in CREC-associated prostatic abscesses.

## Data Availability

The data sets used and/or analyzed during the current study are available from the corresponding author upon reasonable request.
